# Variations in visceral leishmaniasis burden, mortality and the pathway to care within Bihar, India

**DOI:** 10.1186/s13071-017-2530-9

**Published:** 2017-12-07

**Authors:** Sarah Jervis, Lloyd A. C. Chapman, Shweta Dwivedi, Morchan Karthick, Aritra Das, Epke A. Le Rutte, Orin Courtenay, Graham F. Medley, Indranath Banerjee, Tanmay Mahapatra, Indrajit Chaudhuri, Sridhar Srikantiah, T. Déirdre Hollingsworth

**Affiliations:** 1 School of Life Sciences, University of Warwick, Gibbet Hill Campus, Coventry, CV4 7AL UK; 2 CARE India Solutions for Sustainable Development, Patna, Bihar India; 3 Department of Public Health, Erasmus MC, University Medical Center Rotterdam, PO Box 2040, 3000 CA Rotterdam, The Netherlands; 4 London School of Hygiene and Tropical Medicine, Keppel Street, London, WC1E 7HT UK

**Keywords:** Visceral leishmaniasis, Case burden, Bihar, India, Pathway to care, Onset-to-diagnosis time, Onset-to-treatment time, Mortality, Statistical analysis

## Abstract

**Background:**

Visceral leishmaniasis (VL) has been targeted by the WHO for elimination as a public health problem (< 1 case/10,000 people/year) in the Indian sub-continent (ISC) by 2020. Bihar State in India, which accounts for the majority of cases in the ISC, remains a major target for this elimination effort. However, there is considerable spatial, temporal and sub-population variation in occurrence of the disease and the pathway to care, which is largely unexplored and a threat to achieving the target.

**Methods:**

Data from 6081 suspected VL patients who reported being clinically diagnosed during 2012–2013 across eight districts in Bihar were analysed. Graphical comparisons and Chi-square tests were used to determine differences in the burden of identified cases by season, district, age and sex. Log-linear regression models were fitted to onset (of symptoms)-to-diagnosis and onset-to-treatment waiting times to estimate their associations with age, sex, district and various socio-economic factors (SEFs). Logistic regression models were used to identify factors associated with mortality.

**Results:**

Comparisons of VL caseloads suggested an annual cycle peaking in January-March. A 17-fold variation in the burden of identified cases across districts and under-representation of young children (0–5 years) relative to age-specific populations in Bihar were observed. Women accounted for a significantly lower proportion of the reported cases than men (41 vs 59%, *P* < 0.0001). Age, district of residence, house wall materials, caste, treatment cost, travelling for diagnosis and the number of treatments for symptoms before diagnosis were identified as correlates of waiting times. Mortality was associated with age, district of residence, onset-to-treatment waiting time, treatment duration, cattle ownership and cost of diagnosis.

**Conclusions:**

The distribution of VL in Bihar is highly heterogeneous, and reported caseloads and associated mortality vary significantly across different districts, posing different challenges to the elimination campaign. Socio-economic factors are important correlates of these differences, suggesting that elimination will require tailoring to population and sub-population circumstances.

**Electronic supplementary material:**

The online version of this article (10.1186/s13071-017-2530-9) contains supplementary material, which is available to authorized users.

## Background

Visceral leishmaniasis (VL) on the Indian sub-continent (ISC) is a disease caused by the protozoan parasite *Leishmania donovani* and transmitted from human to human by female *Phlebotomus argentipes* sand flies. The symptomatic form of the disease, also known as kala-azar (KA), is characterised by fever, weight-loss and an enlarged liver and spleen, and has a case fatality rate of over 95% if untreated [1]. In recent years, more effective treatments have reduced the case fatality rate to 10% on average [2], with studies suggesting that proximity to a previous VL patient, bed net usage and levels of insecticide spraying are important drivers of VL risk [3–5]. However, the role of delays from onset-of-symptoms to treatment has not been well quantified. The two main strategies for control are improved case detection and management (reductions in onset-to-treatment time), and indoor residual spraying with insecticide.

The ISC has historically suffered the majority of the global burden of VL, with India, Bangladesh and Nepal accounting for 80% of the estimated 200,000–400,000 annual global cases between 2004 and 2008 [2]. However, since 2011 there has been a significant decline in the number of cases in the ISC [6–8]. Consequently, reduction in the incidence of symptomatic VL, to under 1 case/10,000 people/year at sub-district level in the ISC by 2020, is one of the WHO elimination goals. The governments of Bangladesh, India and Nepal have set a more ambitious goal of reaching the elimination target by the end of 2017 [9]. The state of Bihar in northern India is by far the most affected area within the ISC, accounting for 80% of VL cases in India [7] and is still far from elimination with recent estimates of 1–5 cases per 10,000 per year at sub-district (block) level [10, 11].

Over the last 50 years, reported national and regional VL incidence has oscillated in an approximately 15-year cycle [12, 13], with marked declines in recent years [14]. However, there is some evidence of spatial variation in incidence rates [11, 15], the causes of which are poorly understood. Previous studies have found evidence of seasonal variation [3, 12, 16–22], measured by incidence of diagnosis or numbers of sand flies, but the resulting inferences made on VL incidence are complicated by long incubation periods, an uncertain proportion of asymptomatically infected humans and long durations until diagnosis [23]. There have also been few systematic studies of seasonality across multiple areas with different incidence rates. Variations in incidence by both age and sex have also been inferred from many previous studies [3, 5, 12, 21, 22, 24–28], but the majority of these are either single-location studies or based on small numbers of cases. Differences in incidence by location, age and sex have often been hypothesised to be due to differences in access to care, both in terms of individual effects and the indirect effect of long onset-to-diagnosis times on transmission. However, there are few studies of large patient groups investigating drivers of onset-to-diagnosis or treatment patterns [29].

As the VL control programme in Bihar builds towards achieving elimination as a public health problem, and, in the longer term, maintaining this goal and aiming for elimination of transmission, there is a shortage of quantitative information on variation in case burden and drivers of delays in diagnosis. By quantifying these factors across multiple settings, we aim to characterise key sources of variability and inform the design of control programmes to identify and treat the final cases. Using records from 6081 suspected VL patients in eight districts in Bihar, we highlight significant variations in the burden of identified cases (the number of cases identified per head of population per year) and mortality. Although incidence of VL has been the main focus of most previous studies, we additionally study the pathway to care for infected individuals. A large number of symptomatic cases in our dataset, together with the multiple locations and detailed information on socio-economic factors (SEFs) such as housing and cattle ownership, allow us to perform a thorough analysis identifying key differences in VL patients’ pathway to care and odds of survival.

## Methods

### Study population and VL case tracing

CARE India, a non-governmental organization, undertook a rapid situational assessment of VL in 2013 to inform the operation of the kala-azar elimination programme in Bihar. The assessment was conducted as part of the intervention programme funded by the Bill and Melinda Gates Foundation (BMGF) in eight (out of a total of 38) districts of Bihar, including both high and low endemicity districts. Only eight districts were selected due to resource constraints and the need for rapid assessment, and due to CARE India having existing infrastructures for conducting field research in these districts. Due to operational feasibility, the case tracking was limited to symptomatic VL patients only. The reference period for the assessment (the period during which VL diagnosis took place) was January 2012 to June 2013. The following combination of methods was followed to meet this objective (data collection and processing are also described elsewhere [30]):

#### Index case tracing and snowballing

Line lists of VL patients, whose date of diagnosis was within the reference period, reported by the state-run health facilities (block and district hospitals) were compiled. In addition, healthcare facilities belonging to the study area, which specialized in kala-azar care, were contacted to obtain information on potential VL cases. Cases obtained from these two sources were checked to identify and remove duplicates. Attempts were made to trace every patient whose name appeared on the compiled list.

An interview was conducted by a trained study investigator with the successfully traced patient or his/her family members. A medical record review, for patients who possessed any documents about diagnosis and treatment for VL, was conducted along with the interview. Additionally, patient/family members were requested to provide information on any other potential cases of VL in the family or neighbourhood. The contact information of such suspected patients was collected. If a patient could not be traced to the particular address, the case information was shared with other districts in the study, and if the patient could still not be traced, the case was considered untraceable.

Additionally, to increase the sensitivity of the case finding effort, some key informants (such as community health workers and school teachers) from the villages mentioned in the address of potential cases were interviewed to determine if they were aware of any other VL patients or cases of prolonged fever during the reference period, either amongst residents of the same village or elsewhere. The contact information of any such suspected cases was also collected.

#### Mapping and interviewing private healthcare providers

A mapping exercise was undertaken to identify all private laboratories and pharmacists in all villages/towns of each of the study districts. These private healthcare providers were then interviewed to ascertain if they had diagnosed or dispensed medications to any VL patient during the reference period. Moreover, all qualified doctors, and all unqualified practitioners with a large clientele, who were captured through the provider mapping exercise, were contacted to obtain information on any VL patients they had seen or treated (either confirmed or with VL-like symptoms).

The list of potential additional cases generated in this manner was compiled and screened for duplications and repetitions of known cases from government reported lists, and individuals that remained on the non-duplicate list were considered ‘suspected’ VL cases. An attempt was made to trace each suspected case to his or her residence and interview him or her or his/her family, where any documentary proof of diagnosis or treatment was collected. These suspected cases were also asked about any other cases of VL or prolonged fever that they may have known. This iterative process continued until all suspected cases had been interviewed.

All available details of such suspected cases were recorded. No attempt was made to clinically examine suspected cases or confirm their diagnosis through laboratory tests.

The breakdown of the sources via which patients were identified is shown in Table 1. Fourteen percent of cases reported by the national control programme (i.e. those reported by the state-run health facilities) were untraceable. However, approximately 15% of cases identified by the case tracing process were not recorded by the national programme, suggesting that the official figures provide an underestimate of the true VL burden, though much less of an underestimate than suggested by previous studies [31, 32].

**Table 1:** Sources via which VL cases were identified

Primary source via which case was identified	Number	Percentage
Official list from block PHC/SDH/FRU	3835	63.07
Official list from other block PHC/SDH/FRU	231	3.80
Official list from district hospital	1021	16.79
Official list from government medical college	5	0.08
Official list or patient of RMRI	7	0.12
Laboratory	31	0.51
Private hospital/doctor	126	2.07
Chemist/Pharmacist	23	0.38
Another patient	361	5.94
Key informants^a^	388	6.38
Self-informed by patient	53	0.87
Total	6081	100

*Abbreviations*: *PHC* Primary Health Centre, *SDH* Sub-Divisional Hospital, *FRU* First Referral Unit, *RMRI* Rajendra Memorial Research Institute, Patna, Bihar

^a^Key informants: Accredited Social Health Activists (ASHAs), villagers, friends, relatives, school teachers

### VL case definition

A patient was considered to be a case of VL if he/she met any of the following criteria:

(i) If the patient’s name and address were included in the line list of VL patients who were diagnosed by any government facility within the reference period and he/she could be traced to the listed address, regardless of possession of any documents related to diagnosis or treatment.

(ii) If a potential case possessed any documents, from a private or public facility, confirming his/her VL diagnosis (serological test, splenic/bone marrow biopsy) within the study reference period.

(iii) If a potential case possessed documents demonstrating VL treatment, such as prescriptions/pharmacy slips/drug packaging that indicated treatment with miltefosine, sodium stibogluconate (SSG) or amphotericin B, and the start of treatment occurred within the reference period.

### Participant interview

A face-to-face interview was conducted with every eligible VL patient identified using the case detection methodology. If a patient died in the interim, the interview was conducted with the next-of-kin. Data were collected on, among other things, socio-demographic characteristics (age, sex, district), date of onset of symptoms, place and date of diagnosis, pre- and post-diagnosis treatment history, and type and duration of drug treatment. Whenever available, information on treatment and diagnosis history was recorded from medical documents. Data were entered using the Census and Survey Processing System CSPro 5.0 and assessed for logical inconsistencies and to detect and remove duplicate entries.

### Statistical analyses

**Fig. 1:**
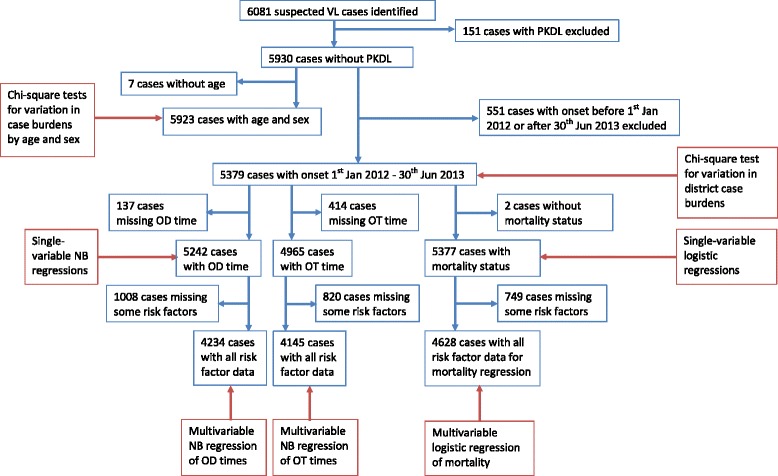
Flowchart for the inclusion of VL cases in statistical analyses. *Abbreviations*: OD, onset-to-diagnosis; OT, onset-to-treatment; NB, negative binomial

Our analyses were conducted using data from the retrospective clinical case finding and patient interviews described above. This dataset consists of information on 6081 suspected VL cases. A flowchart showing the inclusion criteria for VL cases for the statistical analyses is presented in Fig. 1.

#### Burden of identified cases: Seasonal and district-specific variation and age distribution

Unlike some previously reported studies [3, 4] all individuals in the CARE dataset were symptomatic patients. The lack of a control group of disease-free individuals meant that analyses to determine the association of SEFs, such as housing or cattle ownership, with VL risk were not possible. To compare the burdens of identified VL cases in different districts, the expected proportion of VL in each district was computed as being equal to the population proportion (of the eight districts combined), and a Chi-square test was used to judge the collective significance of differences between observed and expected case burdens. Using 2011 census populations by district [33], the sex distribution of cases was analysed following a similar procedure, and the district age distributions of cases were compared. Monthly numbers per district of onsets and diagnoses in 2011–2013 were computed and plotted to examine seasonal variation.

#### Variation in the pathway to care and mortality

The data included information on house construction and size, wall, floor and ceiling materials, cattle ownership and financial contributions to diagnostic tests and treatment. All of these could be viewed as surrogate indicators of economic status, and many could influence the pathway to care. Thus, the effects of variations in housing, cattle ownership and paid-for vs free services on onset-to-diagnosis (OD) and onset-to-treatment (OT) waiting times were analysed together with age, sex and district. As both waiting times have right-skewed distributions with variances much larger than their means (variance/mean = 47.06 days and 38.46 days for the OD and OT times, respectively), negative binomial regression models with exponential link function were used to analyse them. Due to the finite sampling period, there was a bias towards elongated waiting times at the start of the sampling period and shorter waiting times at the end. Therefore we restricted the analysis to the central portion of the data time-period (details in Results). First, single-variable models were fitted to eliminate the least significant relationships, then multivariable models for comparison purposes. Starting with all variables judged significant in single-variable models, factors were accepted or rejected using the likelihood ratio test (LRT). Using a logistic regression model, with a similar selection procedure, we investigated the effects of the same set of factors together with OD and OT waiting times on patients’ risk of death. Although no information as to causes of individuals’ deaths was available, death rates appeared to be both much higher and differently distributed over age and sex amongst patients than in the general Bihar population, such that it seems likely the excess mortality was VL related. To increase understanding of factors influencing overall mortality amongst VL patients, we consequently analysed the risk of dying from any cause during the study period. All regression analyses were performed using Stata 14 [34].

## Results

A total of 6081 suspected VL cases from 131 blocks in eight districts of Bihar were identified in the VL situational assessment. One hundred and fifty-one cases were recorded as having post-kala-azar dermal leishmaniasis (PKDL) and were excluded from all analyses due to uncertainty about whether their illness and treatment data referred to VL or PKDL. Among the 5930 cases remaining, 59% were male, and 41% female; median age was 20, varying from 16 to 30 between districts (see Additional file 1: Table S1 and Additional file 2: Table S2).

### Burden of identified cases

**Fig. 2:**
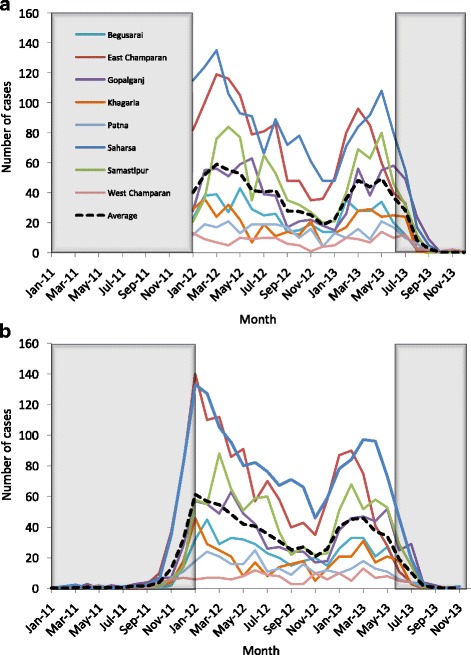
Number of VL diagnoses per month and district by date of diagnosis (**a**) and date of onset of symptoms (**b**). Data were collected on cases diagnosed between January 2012 and June 2013, and therefore incidence outside these periods is marked by a grey box

Monthly counts of diagnoses in January 2012 - June 2013 by district are displayed in Fig. 2a. The monthly numbers of cases by onset for each district have been plotted from the patients’ self-reported onset dates in Fig. 2b. Since inclusion in the study was based on the date of diagnosis being between 1st January 2012 and 30th June 2013, there was an inherent bias towards longer OD times amongst cases with onset prior to 2012, and towards shorter OD times amongst cases with onset after June 2013 (see Additional file 3: Figure S1). Hence, these cases were excluded from the analysis. Patterns of monthly case numbers in most districts in 2012–2013 suggest an annual cycle peaking early in the year (Fig. 2b), though differences between the 2 years’ and eight districts’ maximum and minimum values make it difficult to establish a consistent seasonal pattern.

**Fig. 3:**
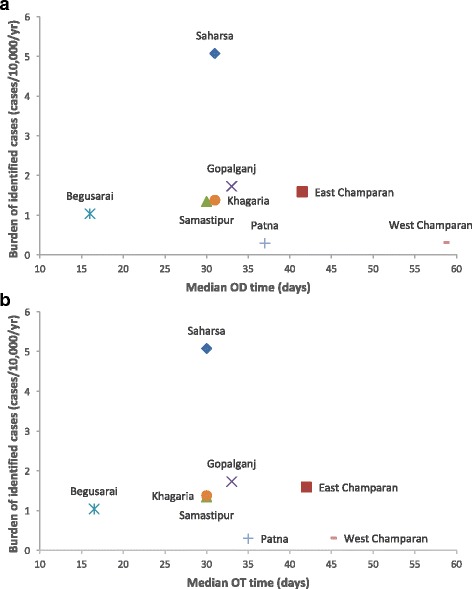
Annual burden of identified cases by district plotted against median onset-to-diagnosis (OD) time (**a**) and median onset-to-treatment (OT) time (**b**)

For comparison of identified case burden between districts, the total population of the blocks in each district with at least one case in January 2012 - June 2013 [35], defined by date of onset, was used to compute the case burden in 2012–2013. Displayed in Table 2 in decreasing order of number of cases, the district burdens of identified cases varied 17-fold from about 1/2000/year to 3/100,000/year. This variation was particularly marked in Saharsa with cases per head almost 3-fold larger than any other district, and Patna and West Champaran, where cases per head were 3-fold lower than any other district. The Chi-square test statistic comparing observed and expected numbers was very large (*χ* ^2^ = 4143.7, *df* = 7, *P* < 0.0001), indicating that relative per-district reported numbers of cases are very different to those expected from the district populations alone. To look for relationships between waiting times and relative case counts, yearly district case burdens in January 2012 - June 2013 (computed as annual number of cases/10,000 of the population) were plotted against median OD and OT times in Fig. 3. Although there was no apparent positive or negative relationship, this does not necessarily mean that reducing waiting times does not lead to significant reductions in case burdens, as there will be a lag before the impact on the case burden is observed. Furthermore, districts that have high case burdens may have shorter OD and OT times due to greater awareness and surveillance of VL, and vice versa for districts with low case burdens.

**Table 2:** Comparison of burden of identified VL cases in eight districts of Bihar, January 2012 - June 2013

District	Total population of affected blocks^a^	Number of VL cases identified	Number of VL cases with onset between Jan 2012 & Jun 2013	Burden of identified cases (cases/10,000/year)	Chi-square statistic
Saharsa	1,900,661	1639	1447	5.075	3012.16
E. Champaran	5,099,371	1383	1214	1.587	38.45
Samastipur	4,261,566	925	862	1.348	0.189
Gopalganj	2,562,012	799	666	1.733	47.28
Begusarai	2,939,764	487	459	1.041	27.49
Khagaria	1,666,886	393	344	1.376	0.418
Patna	5,772,300	289	259	0.299	690.75
W. Champaran	2,776,365	166	128	0.307	326.95
Total	26,978,925	6081	5379	1.329	4143.7^*^

*P-value < 0.0001, *df* = 7, indicating statistically significant difference between identified case burdens and expected case burdens based on district populations

^a^Block populations from 2011 Indian Census [35]

**Fig. 4:**
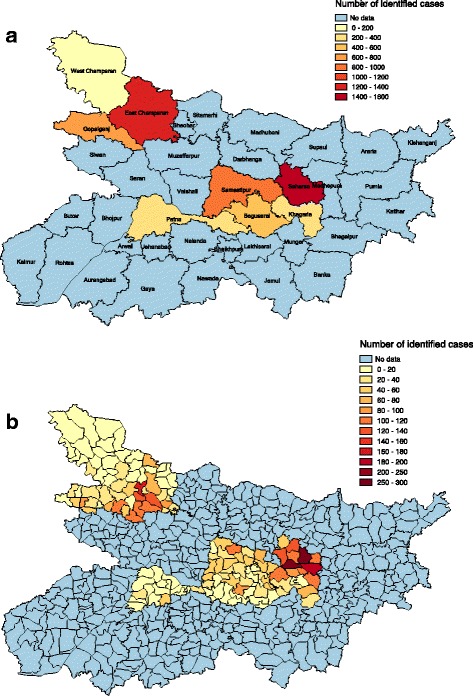
Maps of Bihar showing total numbers of identified VL cases in the eight study districts between January 2012 and June 2013 at district level (**a**) and block level (**b**)

The maps of Bihar in Fig. 4a and b show the total numbers of identified VL cases with onset between January 2012 and June 2013 at the district level and block level, respectively. Equivalent maps for the burden of identified cases at district and block level are provided in Additional file 4: Figure S2. It is clear from these maps that there is considerable spatial heterogeneity in the occurrence of VL. The block-level maps reveal that there is large variation in the identified burden within high-burden districts, with cases per head per year ranging from 1.69/10,000 to over 9/10,000 in blocks in Saharsa and 0.04–6.17/10,000 in East Champaran, with a small number of blocks contributing the majority of the overall burden in these districts. While there appears to be some spatial correlation in reported caseloads at the block level (Additional file 4: Figure S2), there are also blocks with large numbers of identified cases neighbouring blocks with very few cases.

### Age and sex distributions

**Fig. 5:**
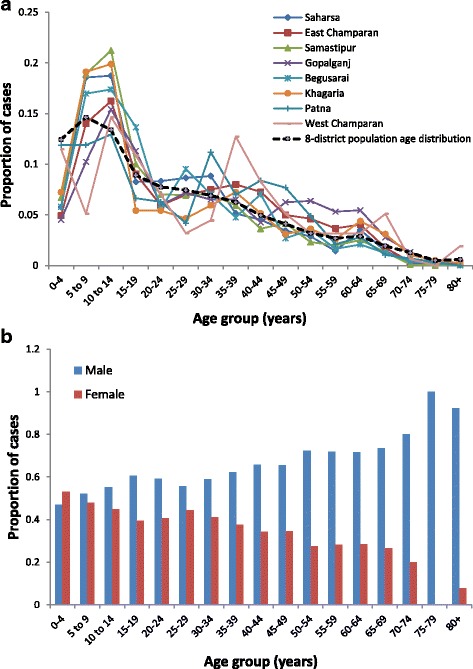
Age distribution of cases. The proportion of cases in 5-year age groups by district (**a**) and proportions of male and female cases in 5-year age groups across all eight districts (**b**)

As illustrated in Fig. 5a, the age distribution of patients did not vary significantly between districts, despite large variability in case counts. For most districts, the proportion of overall cases increases from age 0 to a maximum in 10–14 year-olds, then decreases quickly up to around age 20, and after that decreases more slowly with age. Most districts showed a notable lack of juvenile cases, compared with the corresponding population age distribution. While the cumulative age-distribution of the eight districts shows a general decrease with age, the cases-per-head was only 10.32/100,000 among 0–5 year-olds increasing to 27.81/100,000 in the 10–14 age group, before dipping and then increasing to 26.28/100,000 in the 60–64 age group (see Table 3). A Chi-square test comparing male and female patient numbers of 3501:2422 with population proportions of 14,737,088:13,445,449 gave a *χ* ^2^ statistic of 109 (*df* = 1, *P* < 0.0001), suggesting that men have significantly higher chances of being diagnosed with symptomatic VL than women. Comparing the burden of identified cases by age group for males and females (see Fig. 5b) showed that the under-representation of VL in children was common to both sexes and that the lower rates in females were due to the numbers of reported cases in adult women being reduced relative to men, particularly in older age groups, rates in the 0–14 age range being nearly identical.

**Table 3:** Identified VL case burden by age group

Age group (years)	Population^a^	No. of cases	Ratio (per 100,000)
0–4	3,508,474	362	10.32
5–9	4,123,615	934	22.65
10–14	3,776,045	1050	27.81
15–19	2,516,831	554	22.01
20–24	2,186,709	400	18.29
25–29	2,101,279	431	20.51
30–34	1,949,196	452	23.19
35–39	1,766,119	385	21.80
40–44	1,396,862	327	23.41
45–49	1,156,831	263	22.73
50–54	901,989	228	25.28
55–59	763,541	160	20.95
60–64	818,092	215	26.28
65–69	547,191	109	19.92
70–74	358,440	30	8.37
75–79	140,327	10	7.13
80+	170,996	13	7.60

^a^Population age distribution for the 8 study districts, taken from [33]

### Variation in the pathway to care

**Fig. 6:**
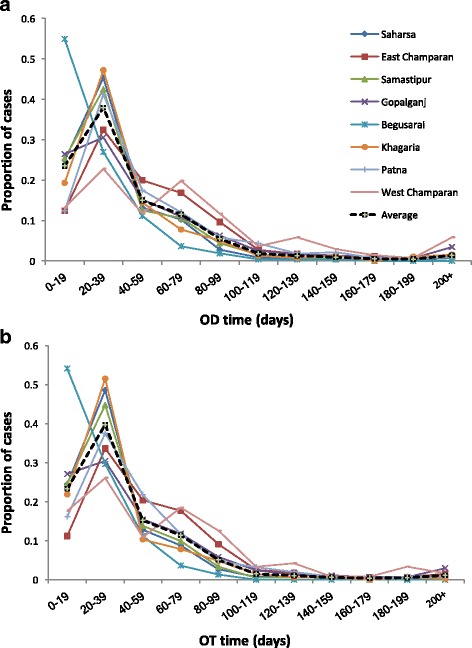
Distributions of onset-to-diagnosis (OD) (**a**) and onset-to-treatment (OT) (**b**) waiting times by district

The district-specific distributions of OD lag, OT lag and treatment durations are illustrated in Fig. 6, and their summary statistics are given in Table 4. Very little difference in treatment duration was observed, with patients in all districts undergoing a median of 4 weeks treatment. OD and OT time varied more, from 16.5 and 17 days in Begusarai (IQR 7–32 and 8–31) to 55 and 45 days (IQR 30.5–80 and 25–90) in West Champaran. Perhaps counterintuitively, patients appeared to wait slightly longer on average for diagnosis than for treatment (median 31 vs 30 days); however, this can be attributed to the delay between clinical examination and diagnostic testing for VL and official confirmation of the VL diagnosis. Treatment for VL, as a high-mortality-rate disease, is likely to have begun shortly after the patient presented with symptoms, but the official diagnosis may not have been recorded until 1 or 2 days later. Additionally, the reliance on self-reported onset dates and illness durations is likely to have introduced some uncertainty into the reported waiting times.

**Table 4:** District-specific summary statistics for onset-to-diagnosis times, onset-to-treatment times and mortality used in regression analyses

	Onset-to-diagnosis time (days) (*n* = 4234)			Onset-to-treatment time (days) (*n* = 4145)			Mortality (*n* = 4628)			
	Mean	Median	IQR	Mean	Median	IQR	Number	Died	% died	% deaths
Saharsa	36.58	31	19–44	34.17	30	20–40	1181	43	3.64	26.06
E. Champaran	51.00	42	28–63	51.22	42	29–61	1136	45	3.96	27.27
Samastipur	37.67	30	19–46	36.52	30	20–45	743	21	2.83	12.73
Gopalganj	47.52	35	18–61	46.70	34	17–60	620	26	4.19	15.76
Begusarai	22.50	16.5	7–32	21.99	17	7–31	356	14	3.93	8.48
Khagaria	37.18	30	20–43	34.43	30	20–38	294	6	2.04	3.64
Patna	48.95	38	27–62	44.54	35	23–59	223	9	4.04	5.45
W. Champaran	59.22	55	30.5–80	59.33	45	25–90	75	1	1.33	0.61
All districts	41.95	31	20–56	40.85	30	20–53	4628	165	3.57	100

Several factors were associated with variation in OD and OT waiting times. The magnitudes of the effects of age, sex, district, housing and diagnosis/treatment facility are displayed in Additional file 5: Table S3 as regression model rate ratios. Waiting times were estimated to increase by 0.4% and 0.5%, respectively, for every year increase in age. OD time was estimated to be up to 3.2 times longer and OT time 2.9 times longer in other districts relative to Begusarai, the district with the shortest average waiting times. Living in a mud-walled house was found to be associated with an 11–12% reduction and stone-walled house with a 6% increase in both waiting times relative to houses with other wall types. Cattle ownership was associated with a 6% increase in OD time while having a house with more than two rooms was estimated to confer a 19–23% increase in waiting times. Paying for diagnosis and being diagnosed at a private centre had near identical effects, with both waiting times appearing to be shorter for individuals choosing to use free services. Being lower-caste was also associated with a 10–11% reduction in waiting times. Patients’ travelling outside their block of residence was estimated to confer a 15–18% increase in their waiting times, while the effect of travelling outside their home district appeared to be smaller but still significant at 12–13%. Relative OD and OT waiting times appeared reduced for 1 or 2 pre-diagnosis treatments (PDTs) and significantly increased for 4 PDTs, with rate ratios rising from 0.53–0.66 for one PDT to 1.21–1.51 for four PDTs. Neither waiting time showed any significant association with gender, house type, roofing or flooring materials, or public vs private treatment.

In the multivariable regressions, house size, cattle ownership, diagnosis cost and public vs private diagnosis were eliminated from both waiting-time models. Treatment cost and same-block diagnosis were not significantly associated with OD, and caste was not significantly associated with OT in the multivariable models (see Table 5 for the optimised models and their covariates’ relative risks and *P*-values). In combination with other factors, the effect of paying for treatment appeared to be reversed, moving from an increase of 6–9% to a reduction of 8% (95% CI 3–13%). Waiting times were still estimated to increase with the number of pre-diagnosis treatments; considerably for OD, with rate ratios from 1.64 (95% CI: 1.22–2.21) for 1 PDT to 3.70 (95% CI: 2.73–5.00) for 4, less markedly for OT, from 1.04 (95% CI: 0.82–1.31) for 2 PDTs to 1.71 (95% CI: 1.35–2.17) for 4. The measures of association for age, district, wall material, caste and travelling for diagnosis remained similar to those observed in the univariate model analyses. When interactions between variables were added to the model, no significant improvement in goodness-of-fit was observed.

**Table 5:** Rate ratios (and 95% confidence intervals) for VL patients’ onset-to-diagnosis (OD) and onset-to-treatment (OT) times for different factors, from multivariable negative-binomial regression models; Bihar, 2012–2013

Variable		Onset-to-diagnosis time (*n* = 4234)			Onset-to-treatment time (*n* = 4145)		
		No. of patients	Rate ratio (95% CI)	*P*-value	No. of patients	Rate ratio (95% CI)	*P*-value
Age	Each 1-year increase	4234	1.003 (1.002–1.004)	< 10^−4^	4145	1.003 (1.002–1.004)	< 10^−4^
District^a^	Saharsa	956	1.99 (1.82–2.18)	< 10^−4^	927	1.94 (1.77–2.12)	< 10^−4^
	E. Champaran	1108	2.26 (2.07–2.47)	< 10^−4^	1102	2.33 (2.14–2.54)	< 10^−4^
	Samastipur	710	2.17 (1.97–2.39)	< 10^−4^	682	2.16 (1.97–2.38)	< 10^−4^
	Gopalganj	572	2.22 (2.01–2.45)	< 10^−4^	567	2.28 (2.07–2.51)	< 10^−4^
	Begusarai	326	–	–	318	–	–
	Khagaria	291	1.61 (1.44–1.80)	< 10^−4^	277	1.56 (1.40–1.74)	< 10–4
	Patna	195	1.85 (1.63–2.10)	< 10^−4^	194	1.76 (1.55–1.99)	< 10–4
	W. Champaran	76	2.75 (2.30–3.27)	< 10^−4^	78	2.74 (2.29–3.28)	< 10–4
Wall^b^	Grass + straw	2034	1.05 (0.97–1.12)	0.220	1994	1.07 (0.99–1.14)	0.077
	Mud	478	–	–	469	–	–
	Mud, stone	812	1.10 (1.02–1.20)	0.014	787	1.14 (1.06–1.23)	0.001
	Concrete	910	1.004 (0.93–1.09)	0.916	895	1.01 (0.94–1.09)	0.752
No. of pre-diagnosis treatments	0	23	–	–	39	–	–
	1	1585	1.64 (1.22–2.21)	< 10^−4^	1548	0.78 (0.61–0.98)	0.032
	2	1452	2.18 (1.62–2.94)	< 10^−4^	1407	1.04 (0.82–1.31)	0.758
	3	748	2.99 (2.22–4.03)	< 10^−4^	736	1.37 (1.09–1.74)	0.008
	4	426	3.70 (2.73–5.00)	< 10^−4^	415	1.71 (1.35–2.17)	< 10^−4^
Lower caste	No	2943	–	–	–	–	–
	Yes	1291	0.95 (0.91–0.99)	0.029	–		
Treatment cost	Free	–	–	–	3379	–	–
	Paid	–	–	–	766	0.92 (0.87–0.97)	0.003
Same-district diagnosis	No	332	–	–	321	–	–
	Yes	3902	0.92 (0.85–0.99)	0.037	3824	0.90 (0.83–0.98)	0.011
Same-block diagnosis	No	–	–	–	1850	–	–
	Yes	–	–	–	2295	0.95 (0.905–0.995)	0.032

^a^Baseline is Begusarai as district with shortest average OD and OT waiting times

^b^Baseline is mud-walled housing as housing type with shortest average OT waiting time

### Factors influencing mortality rates

Analysis of age- and sex-specific death rates among VL patients with onset of symptoms in 2012 revealed that for 0–14 year-olds mortality was higher among females than males, with the pattern reversed in adults (see Table 6). Even though all patients within the study were assumed to have received treatment, death rates were still much higher in all age groups and both sexes amongst patients than the general Bihar population (apart from women over the age of 60) in 2012 [36], and showed different patterns with age and sex more closely resembling the age-sex distribution of VL cases. This suggests that VL patients had an elevated risk of dying, as noticed in previous studies [37], and factors underlying this were thus worthy of investigation.

**Table 6:** Comparison of age-specific death rates in VL patients and in Bihar population

Age group	VL patients (2012)			Deaths (2012)			Deaths per 1000 VL cases per year			Deaths per 1000 general population per year (2012)^a^		
	Male	Female	Total	Male	Female	Total	Male	Female	Total	Male	Female	Total
0–4	84	116	200	2	8	10	23.81	68.97	50.00	11.2	11.8	11.5
5–14	649	556	1205	5	18	23	7.70	32.37	19.09	0.9	0.8	0.9
15–59	1234	777	2011	60	32	92	48.62	41.18	45.75	3.2	2.6	2.9
60+	174	51	225	26	3	29	149.43	58.82	128.89	42.9	49.0	45.7

^a^From [36]

Comparison of *P*-values and log-likelihoods for univariate logistic models indicated that sex, all housing factors, free vs paid-for treatment, public vs private facilities, caste, the number of pre-diagnosis treatments, and diagnosis within vs without block- and district-of-residence had no significant association with risk of death. By backwards selection starting from a model incorporating age, district, waiting times, cattle ownership and free vs paid-for diagnostic test, death risk was subsequently shown to have no significant association with OD time (see Table 7 for the optimised model and the covariates’ relative risks and *P*-values). The greatest risk-reducer appeared to be cattle ownership, conferring a 39.4% reduction, and free diagnosis was associated with a more than two-fold increase in mortality risk, with an odds ratio of 2.19, although the confidence intervals (CIs) of both odds ratios were quite wide at 0.44–0.85 and 1.49–3.21 respectively. Each additional year of age was associated with a 3% increase in risk (95% CI: 2.5–4.0%) and each extra day waiting for treatment conferred a small but significant 0.6% increase (95% CI: 0.2–0.9%). Although there are possible confounding factors, death risk was estimated to decrease by 9% per day of treatment (95% CI: 7–11%).

**Table 7:** Odds ratios (and 95% confidence intervals) for VL patients’ risk of death for different factors, from multivariable logistic regression model; Bihar, 2012–2013 (*n* = 4628)

Variable		No. of patients		Odds ratio	95% CI	*P*-value
		Total	Dead			
Age	Each 1-year increase	4628	165	1.03	1.025–1.04	< 10^−4^
District	Saharsa	1181	43	2.65	1.07–6.57	0.036
	E. Champaran	1136	45	2.60	1.04–6.48	0.040
	Samastipur	743	21	1.58	0.60–4.15	0.357
	Gopalganj	620	26	2.96	1.11–7.85	0.029
	Begusarai	356	14	3.22	1.16–8.91	0.025
	Khagaria	294	6	–	–	–
	Patna	223	9	1.85	0.62–5.53	0.272
	W. Champaran	75	1	0.71	0.08–6.18	0.754
Onset-to-treatment time	Each 1-day increase	4628	165	1.006	1.002–1.009	0.002
Treatment duration	Each 1-day increase			0.91	0.89–0.93	< 10^−4^
Cattle owner	No	1767	79	–	–	–
	Yes	2861	86	0.61	0.44–0.85	0.004
Paid for test	Paid	1980	53	–	–	–
	Free	2648	112	2.19	1.49–3.21	< 10^−4^

Forward selection exploring models with pairwise interactions between district and other factors found no significant variation in associations between districts. Similarly, models incorporating interactions between multiple housing-material factors did not fit significantly better than those without interactions.

## Discussion

This study is one of few to assess variation in VL case burden in Bihar and the first to examine the spatial differences and effects of socio-economic factors (SEFs) on VL mortality rates, diagnosis and treatment in the ISC, highlighting important variability in the pathway to care.

### Burden of identified cases: Spatial, seasonal, sex- and age-specific variations

Although calculation of district-specific incidences was not possible due to insufficient knowledge of the population- and person-time- at-risk, comparing case counts of some districts with their respective populations shows that there are significant differences in their burdens of identified cases. The districts with the highest and lowest numbers of reported cases were Saharsa and West Champaran respectively, despite the latter having the larger population. There is also strong spatial heterogeneity in the numbers and burdens of identified cases at the block level, with greater variation in block burdens than district burdens.

At state-level there has been a significant decline in the number of reported VL cases in Bihar since 2011 (from 25,222 cases in 2011 to 4773 cases in 2016) [7], and in all districts in this study apart from West Champaran the total number of identified cases decreased between January-June 2012 and January-June 2013 (Fig. 2b). There are various factors that may have contributed to this decrease. One is greater availability and awareness of free diagnosis and treatment in government health centres [38] and shorter times to treatment (cf. Table 4 with Table 1 in [39]), which may have led to significant reductions in transmission by shortening the periods for which VL cases are infectious. Another is reported increased coverage of indoor residual insecticide spraying (IRS) [28, 40], which may have reduced transmission by reducing sand fly densities. However, whether IRS coverage in the eight study districts increased before 2012 is uncertain, and there is evidence of sub-standard IRS implementation and widespread sand fly resistance to DDT (dichlorodiphenyltrichloroethane, the insecticide used at the time) in Bihar [41–43]. Given the long-term cycles in reported case numbers [12, 13], it is also possible that much of the decrease is due to long-lasting immunity having built up in populations affected during the previous epidemic in the late 2000s, such that the pool of susceptible individuals who can become infected and develop VL has been depleted [44].

The annual cycle observed in 2012–2013 monthly onsets and, to a lesser extent, in monthly diagnosis numbers, is almost certainly attributable to seasonal variation in sand fly populations. The majority of previous studies of seasonal variation in sand fly numbers found the vector density to be positively correlated with temperature and negatively correlated with rainfall [16–19]. Peak populations were recorded in summer and after the end of the rainy season, although with variations (March-April and November [16], May-July and October-November [17], March-May [18], and June-July and November [19]). The summer (March-May) peak appears to coincide with that seen both in the monthly onsets in this study and in other studies examining VL seasonality, which found cases to peak in March-April [20, 22]. This is unexpected; given an infected-symptomatic sojourn time of 2–5 months [3, 15, 23, 45], one would expect cases to peak later in the year than sand fly density, and indeed some studies have observed highest numbers of VL cases in July-September or April-June and lowest numbers in January-March [3, 12, 18, 21]. However, the peak in cases in March - April fits with the peak in sand fly abundance after the rainy season (October-November) in the previous year. One possible explanation for the lack of a second case peak after the summer sand fly peak could be that the proportion of infective sand flies only peaks once, late in the year [46]. Variation in the timing and appearance of the case peak is likely to be due to the considerable variation in the incubation period for disease (since longer incubation periods dampen the seasonality in the number of cases from that in the sand fly abundance [47]). However, the previous studies suggesting a later peak incidence than our analyses had some weaknesses as ascertainers of seasonality. The studies by Bern and Perry et al. [3, 21] were both cross-sectional studies with relatively low numbers of actual symptomatic VL patients, and the historical case counts used by Bora [12] are likely to be numbers of diagnoses rather than onsets, which could easily explain the relative delay in peak cases via the added OD waiting times. Although both onset and diagnosis numbers in our data show an annual cycle, the oscillation is much less clear in the monthly diagnosis numbers. This adds uncertainty to the seasonality reported by Bora [12] and suggests that added variation from factors such as waiting times can easily distort or cloud the results of diagnostic tests as a representation of temporal VL distribution. Thus, future studies examining symptomatic VL epidemiology might be better using patients’ records of their disease progression, as used here, rather than clinical test results, even accounting for the greater measurement errors associated with self-reported data. To overcome the uncertainty about the relationship between seasonally varying sand fly and case numbers, the two need to be measured simultaneously and a better understanding of asymptomatic sojourn times is needed.

As illustrated in Fig. 5a and Table 3, young children are noticeably less affected by VL relative to the age distribution of the general population. Although this is in contrast to the study by Perry et al. [21] which estimated the 0–10 age group to be at highest risk and the 11–20 age group at lowest risk of VL, Perry et al. [21] only included 45 symptomatic VL cases and a reduced incidence of VL in young children has been observed in the majority of previous studies [3, 5, 12, 22, 24, 27, 28]. If the apparently low burden of cases in under-fives does reflect the actual underlying age distribution of disease, a possible explanation is variation in sand fly exposure: via different patterns of daytime movement or prioritisation of bed nets, young children have lower levels of VL infection because they are less frequently bitten by sand flies. Another hypothesis for under-representation is under-reporting of symptomatic VL in children: in regions where there are many infant deaths due to fever, many fatal VL cases in infants could have been passed over when collecting our dataset. This is suggested by Bihar population surveys reviewed by Bora [12], where the 1989 number of cases in the 0–9 age group is 8.6% higher than that in 1979; given that the population-wide case counts are similar in the 2 years, it is possible that an apparent increase in children is due to a reduction in under-reporting rather than an actual change in age-specific levels due to the long-term dynamics of the disease. It is also possible that there is little variation by age in actual biting and infection rates, but children have a lower probability of an asymptomatic infection developing into clinical VL, so are consequently rarer in the symptomatic population.

The hypotheses of lower exposure of susceptible individuals or lower probability of infected individuals becoming symptomatic could also account for the apparent shortfall in VL cases among women relative to men, also reported by previous studies [3, 5, 12, 21, 22, 24–27, 48]. Another possible explanation is under-reporting of female cases due to women having, on average, poorer access to appropriate healthcare. This hypothesis is supported by Alvar et al. [49], where 60–80% of health facility patients were male while sex ratios were almost equal in population-based studies. This theory could also explain why only adult women appear to have reduced risk: before the age of 15, say, males and females are equally likely for their parents/guardians to take them to a doctor when unwell, while adult women are less able than men to travel to healthcare facilities. Given that our analyses of factors affecting waiting times for diagnosis and treatment (discussed in more detail below) found no significant difference between sexes, it would seem probable that reduced access to healthcare for women is more likely to have manifest as a lack of, rather than a delay in, diagnosis and treatment for some symptomatically infected women. The apparent widening of the gap between the numbers of identified cases in men and in women with age in our data is strikingly similar to that observed in a study of 8749 VL patients from the Vaishali district of Bihar [22], and emphasises the need for further investigation of the factors affecting reported numbers of male and female cases.

### Significant differences in the pathway to care

In our analyses of waiting times, we found significant differences between districts even after the inclusion of many SEFs one might expect to account for such variation. Given that several SEFs found to be significant in univariate regression models were subsequently excluded from multivariate models, this could be because an individual’s district is a more informative (summary) measure of their effective socio-economic status than any available single SEF and as such, the spatial association might be replaceable by the right combination of SEFs. Alternatively, the observed association might be attributable to unmeasured variation in health provision between districts; this could include healthcare staffing levels and VL awareness, as well as documented statistics such as relative availability of public health centres. Out of the other factors judged to have significant effects, having more PDTs or having to travel outside one’s residential area were unsurprisingly estimated to increase both waiting times. Somewhat unexpectedly, paying for treatment appeared to decrease OT waiting times only when in combination with other variables, and lower-caste individuals were estimated to have shorter diagnosis and treatment waiting times on average. The apparent reduction in waiting times for lower-caste individuals is likely also due to a difference in awareness; as VL is a disease associated with poverty, clinical practitioners might be more likely to VL-test and medicate a lower-caste patient with a fever.

### Mortality

Another important issue for minimising the effects of VL as a public health problem is the reduction of mortality related to the disease. To develop a strategy for this, it is necessary to understand which factors significantly influence mortality among patients. Comparing the effects of numerous factors on an individual’s probability of death using logistic regression models, somewhat surprisingly no significant difference was found between sexes or different housing types and materials. The increase in mortality with age was unsurprising, as was the small but significant increase with waiting-time to treatment. The associations between increased treatment duration and cattle ownership and lower mortality are more difficult to interpret: the former might be biased by some individuals only receiving longer treatments because they survived the pre- or early-treatment stage, while cattle ownership might be directly protective via better nutrition but alternatively could just be a marker of higher average socio-economic status. The lower mortality among patients paying for diagnosis could be wealth-related: higher-income individuals might be more likely to pay for care, or facilities providing free diagnosis tests might be more accessible in poorer areas, with differences in mortality thus attributable to wealth-associated differences in average baseline health. Due probably to the low numbers of VL patients and thus even lower number of patient deaths observable from case-control studies, there appear to be very few previous studies examining factors affecting mortality rates amongst VL patients. Barnett et al. [25] and Huda et al. [50] both reported differences in male and female mortality rates, whereas sex was not found to significantly affect death risk in our regression analyses. However, these two studies suggested opposing results - 33 vs 75% of deaths among males - and were based on very low numbers of deaths–8 and 9, respectively. Similarly to our analyses, a recent study using the same data source, by Das et al. [30] found cattle ownership and shorter onset-to-diagnosis waiting times to be associated with a reduced death risk and age and private treatment associated with an increased risk, but gender to have little effect. Although the results in Das et al. [30] also suggested that house type and caste affected mortality while these were eliminated from our preferred model, this is likely due to a difference in methods; Das et al. measured variations in death risk over time using Cox proportional hazard models while our analyses were of the total hazard of dying. Consequently, it could be that caste and house type have a significant effect on time-dependent risk of death but not the overall risk. Alternatively, the inclusion of district in our regression analyses might have accounted for variations in mortality attributed to housing or caste in [30].

Our analyses did have several limitations. The lack of information on disease-free individuals prevented any investigation of the effects of SEFs on VL incidence across Bihar. The use of retrospective questionnaires for data collection meanwhile meant that records of both key dates along the pathway to care and some SEFs were incomplete and sometimes contradictory, while the records of deaths within the study did not differentiate between deaths caused by VL and those from other causes. Nevertheless, we were able to gain valuable insight into the effects of housing, cattle and location on VL diagnosis and treatment as well as variations in the distribution of the disease itself.

## Conclusions

We conclude that in various VL-endemic districts of Bihar there remain challenges in the elimination of VL and that there is considerable variation between regions in the pathway to care. For long-term control, more attention needs to be focused on districts with a high case burden, and efforts should be made in all regions to reduce waiting times for diagnosis and treatment, either by increasing provision or awareness, and to improve healthcare access for women.

## Additional files

Additional file 1: Table S1. Distribution of socio-economic factors across the eight study districts. (DOCX 30 kb)

Additional file 2: Table S2. Distribution of continuous variables (age, waiting times and house size) for the eight study districts. (DOCX 15 kb)

Additional file 3: Figure S1. Box-plots of distribution of onset-to-diagnosis waiting times by season of onset. (DOCX 90 kb)

Additional file 4: Figure S2. Maps of Bihar showing burdens of identified cases in study districts for January 2012 - June 2013 at (**a**) district level and (**b**) block level. (DOCX 996 kb)

Additional file 5: Table S3. Single-variable negative-binomial regression models for OD and OT waiting times. (DOCX 23 kb)

## Abbreviations

CI: Confidence interval
IQR: Interquartile range
ISC: Indian sub-continent
KA: Kala-azar
LRT: Likelihood ratio test
OD: Onset-to-diagnosis
OT: Onset-to-treatment
PKDL: Post-kala-azar dermal leishmaniasis
SEF: Socio-economic factor
VL: Visceral leishmaniasis
