# Supplementary material for: Variations in visceral leishmaniasis burden, mortality and the pathway to care within Bihar, India
Source: Parasit Vectors. 2017 Dec 7;10:601. doi: 10.1186/s13071-017-2530-9 (PMC5719561; doi:10.1186/s13071-017-2530-9)
Supplement: Supplementary file 1 — Distribution of socio-economic factors across the eight study districts. (DOCX 30 kb) [file 13071_2017_2530_MOESM1_ESM.docx]

**Additional file 1: Table S1.** Distribution of socio-economic factors across the eight study districts.

| Variable | | Saharsa | | E. Champaran | | Samastipur | | Gopalganj | | Begusarai | | Khagaria | | Patna | | W. Champaran | | Total | |
| --- | --- | --- | --- | --- | --- | --- | --- | --- | --- | --- | --- | --- | --- | --- | --- | --- | --- | --- | --- |
|  |  | n | % | n | % | n | % | n | % | n | % | n | % | n | % | n | % | n | % |
| Sex | Male | 898 | 56.3 | 833 | 61.2 | 518 | 57 | 458 | 60.9 | 290 | 60 | 223 | 57.6 | 183 | 64 | 100 | 63.7 | 3503 | 59.1 |
|  | Female | 697 | 43.7 | 528 | 38.8 | 391 | 43 | 294 | 39.1 | 193 | 40 | 164 | 42.4 | 103 | 36 | 57 | 36.3 | 2427 | 40.9 |
| House | Kachcha | 1308 | 82.1 | 1023 | 75.2 | 509 | 56 | 278 | 37 | 332 | 68.7 | 284 | 76.1 | 155 | 54.2 | 101 | 64.3 | 3990 | 67.5 |
|  | Semi-pucca | 229 | 14.4 | 250 | 18.4 | 298 | 32.8 | 260 | 34.6 | 128 | 26.5 | 60 | 16.1 | 79 | 27.6 | 30 | 19.1 | 1334 | 22.6 |
|  | Pucca | 56 | 3.5 | 88 | 6.5 | 102 | 11.2 | 214 | 28.5 | 23 | 4.8 | 29 | 7.8 | 52 | 18.2 | 26 | 16.6 | 590 | 10 |
| Wall | Grass+straw | 754 | 49.7 | 779 | 59.1 | 391 | 44.9 | 189 | 26.5 | 170 | 37.5 | 213 | 60.7 | 24 | 9.6 | 71 | 53.8 | 2591 | 46.2 |
|  | Mud | 292 | 19.2 | 66 | 5 | 96 | 11 | 31 | 4.3 | 75 | 16.6 | 16 | 4.6 | 98 | 39 | 7 | 5.3 | 681 | 12.1 |
|  | Mud+stone | 284 | 18.7 | 249 | 18.9 | 239 | 27.4 | 124 | 17.4 | 94 | 20.8 | 58 | 16.5 | 47 | 18.7 | 13 | 9.8 | 1108 | 19.8 |
|  | Concrete | 188 | 12.4 | 225 | 17.1 | 145 | 16.6 | 369 | 51.8 | 114 | 25.2 | 64 | 18.2 | 82 | 32.7 | 41 | 31.1 | 1228 | 21.9 |
| Ceiling | Thatch | 852 | 56.1 | 753 | 57.1 | 418 | 48 | 209 | 29.4 | 179 | 39.5 | 194 | 55.3 | 87 | 34.7 | 73 | 55.3 | 2765 | 49.3 |
|  | Concrete | 149 | 9.8 | 194 | 14.7 | 115 | 13.2 | 312 | 43.8 | 99 | 21.9 | 51 | 14.5 | 77 | 30.7 | 37 | 28 | 1034 | 18.4 |
|  | Earth tile | 400 | 26.3 | 201 | 15.2 | 292 | 33.6 | 26 | 3.7 | 154 | 34 | 59 | 16.8 | 67 | 26.7 | 5 | 3.8 | 1204 | 21.5 |
|  | Asbestos | 118 | 7.8 | 171 | 13 | 45 | 5.2 | 165 | 23.2 | 21 | 4.6 | 47 | 13.4 | 20 | 8 | 17 | 12.9 | 604 | 10.8 |
| Floor | Mud | 1455 | 96 | 1271 | 96.9 | 842 | 97.1 | 623 | 87.4 | 426 | 94 | 339 | 96.9 | 232 | 92.4 | 120 | 90.9 | 5308 | 94.9 |
|  | Concrete | 60 | 4 | 40 | 3.1 | 25 | 2.9 | 90 | 12.6 | 27 | 6 | 11 | 3.1 | 19 | 7.6 | 12 | 9.1 | 284 | 5.1 |
| Rooms | 1-2 | 1380 | 91.3 | 839 | 63.7 | 793 | 92 | 472 | 66.2 | 411 | 90.9 | 312 | 89.4 | 152 | 60.6 | 83 | 62.9 | 4442 | 79.5 |
|  | >2 | 132 | 8.7 | 478 | 36.3 | 69 | 8 | 241 | 33.8 | 41 | 9.1 | 37 | 10.6 | 99 | 39.4 | 49 | 37.1 | 1146 | 20.5 |
| Cattle | Yes | 963 | 60.4 | 933 | 68.6 | 480 | 52.9 | 528 | 70.2 | 224 | 46.5 | 199 | 53.5 | 164 | 57.3 | 101 | 64.7 | 3592 | 60.8 |
|  | No | 631 | 39.6 | 428 | 31.4 | 428 | 47.1 | 224 | 29.8 | 258 | 53.5 | 173 | 46.5 | 122 | 42.7 | 55 | 35.3 | 2319 | 39.2 |
| Test cost | Free | 1114 | 74 | 669 | 51 | 524 | 60.4 | 131 | 18.4 | 264 | 58.5 | 237 | 68.1 | 177 | 70.5 | 70 | 54.3 | 3186 | 57.1 |
|  | Paid | 391 | 26 | 642 | 49 | 344 | 39.6 | 581 | 81.6 | 187 | 41.5 | 111 | 31.9 | 74 | 29.5 | 59 | 45.7 | 2389 | 42.9 |
| Treatment cost | Free | 1113 | 84.1 | 1107 | 85.7 | 695 | 84.1 | 410 | 59.4 | 317 | 81.7 | 279 | 82.8 | 202 | 85.2 | 86 | 72.9 | 4209 | 80.8 |
|  | Paid | 210 | 15.9 | 185 | 14.3 | 131 | 15.9 | 280 | 40.6 | 71 | 18.3 | 58 | 17.2 | 35 | 14.8 | 32 | 27.1 | 1002 | 19.2 |
| Diagnosis centre | Public | 1223 | 81 | 781 | 59.8 | 583 | 67.6 | 158 | 22.3 | 256 | 58 | 264 | 75.9 | 181 | 72.1 | 88 | 68.8 | 3534 | 63.6 |
|  | Private | 287 | 19 | 526 | 40.2 | 280 | 32.4 | 552 | 77.7 | 185 | 42 | 84 | 24.1 | 70 | 27.9 | 40 | 31.3 | 2024 | 36.4 |
| Treatment centre | Public | 1333 | 89.2 | 1235 | 94.3 | 704 | 84.2 | 479 | 67.7 | 358 | 82.5 | 291 | 85.3 | 200 | 81 | 93 | 73.8 | 4693 | 85.4 |
|  | Private | 161 | 10.8 | 75 | 5.7 | 132 | 15.8 | 229 | 32.3 | 76 | 17.5 | 50 | 14.7 | 47 | 19 | 33 | 26.2 | 803 | 14.6 |
| Pre-diagnosis treatment | 0 | 77 | 4.8 | 47 | 3.5 | 61 | 6.7 | 41 | 5.5 | 30 | 6.2 | 38 | 9.8 | 35 | 12.2 | 50 | 31.8 | 379 | 6.4 |
|  | 1 | 780 | 48.9 | 334 | 24.5 | 520 | 57.2 | 301 | 40 | 114 | 23.6 | 39 | 10.1 | 17 | 5.9 | 32 | 20.4 | 2137 | 36 |
|  | 2 | 549 | 34.4 | 440 | 32.3 | 235 | 25.9 | 200 | 26.6 | 202 | 41.8 | 159 | 41.1 | 63 | 22 | 31 | 19.7 | 1879 | 31.7 |
|  | 3 | 146 | 9.2 | 298 | 21.9 | 74 | 8.1 | 125 | 16.6 | 105 | 21.7 | 103 | 26.6 | 97 | 33.9 | 24 | 15.3 | 972 | 16.4 |
|  | 4 | 43 | 2.7 | 242 | 17.8 | 19 | 2.1 | 85 | 11.3 | 32 | 6.6 | 48 | 12.4 | 74 | 25.9 | 20 | 12.7 | 563 | 9.5 |
| Caste | Upper | 1011 | 63.7 | 1018 | 75.2 | 624 | 69.6 | 602 | 80.9 | 325 | 71.1 | 239 | 63.4 | 139 | 49.1 | 115 | 73.2 | 4073 | 69.6 |
|  | Lower | 575 | 36.3 | 336 | 24.8 | 272 | 30.4 | 142 | 19.1 | 132 | 28.9 | 138 | 36.6 | 144 | 50.9 | 42 | 26.8 | 1781 | 30.4 |
| Same-district diagnosis | No | 27 | 2 | 77 | 6 | 77 | 9 | 105 | 15.1 | 99 | 22 | 40 | 11.8 | 15 | 6 | 21 | 16.9 | 461 | 8.6 |
|  | Yes | 1353 | 98 | 1214 | 94 | 779 | 91 | 591 | 84.9 | 350 | 78 | 300 | 88.2 | 236 | 94 | 103 | 83.1 | 4926 | 91.4 |
| Same-block diagnosis | No | 259 | 20.1 | 879 | 68.2 | 321 | 39 | 378 | 56.3 | 207 | 46.9 | 67 | 19.9 | 119 | 52.2 | 45 | 37.2 | 2275 | 43.8 |
|  | Yes | 1027 | 79.9 | 409 | 31.8 | 503 | 61 | 294 | 43.8 | 234 | 53.1 | 269 | 80.1 | 109 | 47.8 | 76 | 62.8 | 2921 | 56.2 |
