# Supplementary material for: Variations in visceral leishmaniasis burden, mortality and the pathway to care within Bihar, India
Source: Parasit Vectors. 2017 Dec 7;10:601. doi: 10.1186/s13071-017-2530-9 (PMC5719561; doi:10.1186/s13071-017-2530-9)
Supplement: Supplementary file 2 — Distribution of continuous variables (age, waiting times and house size) for the eight study districts. (DOCX 15 kb) [file 13071_2017_2530_MOESM2_ESM.docx]

**Additional file 2: Table S2.** Distribution of continuous variables (age, waiting times and house size) for the eight study districts, for cases with onset between January 2012 and June 2013.

|  | | | | | | | | | | | | | | | |
| --- | --- | --- | --- | --- | --- | --- | --- | --- | --- | --- | --- | --- | --- | --- | --- |
|  | Age (years) | | | Onset-to-diagnosis (days) | | | Onset-to-treatment (days) | | | Treatment duration (days) | | | Number of rooms | | |
|  | Mean | Median | IQR | Mean | Median | IQR | Mean | Median | IQR | Mean | Median | IQR | Mean | Median | IQR |
| Saharsa | 22.51 | 18 | 10-30 | 37.68 | 31 | 19-42 | 33.57 | 30 | 19-40 | 28.84 | 28 | 28-28 | 1.67 | 2 | 1-2 |
| E. Champaran | 26.27 | 22 | 10-40 | 50.92 | 41.5 | 28-63 | 50.85 | 42 | 29-60 | 29.09 | 28 | 28-28 | 2.59 | 2 | 2-3 |
| Samastipur | 21.45 | 16 | 9-30 | 38.41 | 30 | 19-49 | 36.82 | 30 | 20-45 | 27.30 | 28 | 28-28 | 1.67 | 2 | 1-2 |
| Gopalganj | 28.98 | 25 | 12-45 | 46.41 | 33 | 17-60 | 45.79 | 33 | 17-60 | 29.61 | 28 | 28-30 | 2.71 | 2 | 2-3 |
| Begusarai | 22.25 | 17 | 10-30 | 23.15 | 16 | 7-32 | 21.86 | 16.5 | 8-31 | 30.29 | 28 | 28-30 | 1.57 | 1 | 1-2 |
| Khagaria | 24.20 | 16 | 8-38 | 43.06 | 31 | 21-48 | 35.28 | 30 | 20-40 | 29.21 | 28 | 28-28 | 1.64 | 2 | 1-2 |
| Patna | 25.54 | 25 | 10-40 | 50.25 | 37 | 27-61 | 44.45 | 35 | 23-57 | 27.94 | 28 | 28-30 | 2.77 | 2 | 2-3 |
| W.Champaran | 29.59 | 30 | 12-42.5 | 74.70 | 58.5 | 30.5-92 | 62.57 | 45 | 25-90 | 29.71 | 28 | 28-30 | 2.58 | 2 | 1-3 |
| Total | n = 5375 | | | n = 5242 | | | n = 4965 | | | n = 4866 | | | n = 5102 | | |
|  | 24.4 | 20 | 10-35 | 41.82 | 31 | 20-54 | 39.91 | 30 | 20-50 | 28.86 | 28 | 28-28 | 2.07 | 2 | 1-2 |
