# Supplementary material for: Variations in visceral leishmaniasis burden, mortality and the pathway to care within Bihar, India
Source: Parasit Vectors. 2017 Dec 7;10:601. doi: 10.1186/s13071-017-2530-9 (PMC5719561; doi:10.1186/s13071-017-2530-9)
Supplement: Supplementary file 3 — Box-plots of distribution of onset-to-diagnosis waiting times by season of onset. (DOCX 90 kb) [file 13071_2017_2530_MOESM3_ESM.docx]

**Additional file 3:** **Figure S1.** Distribution of onset-to-diagnosis (OD) waiting times by season of onset, showing bias towards longer OD times at start of study (before January 2012) and shorter OD times at end (after June 2013)


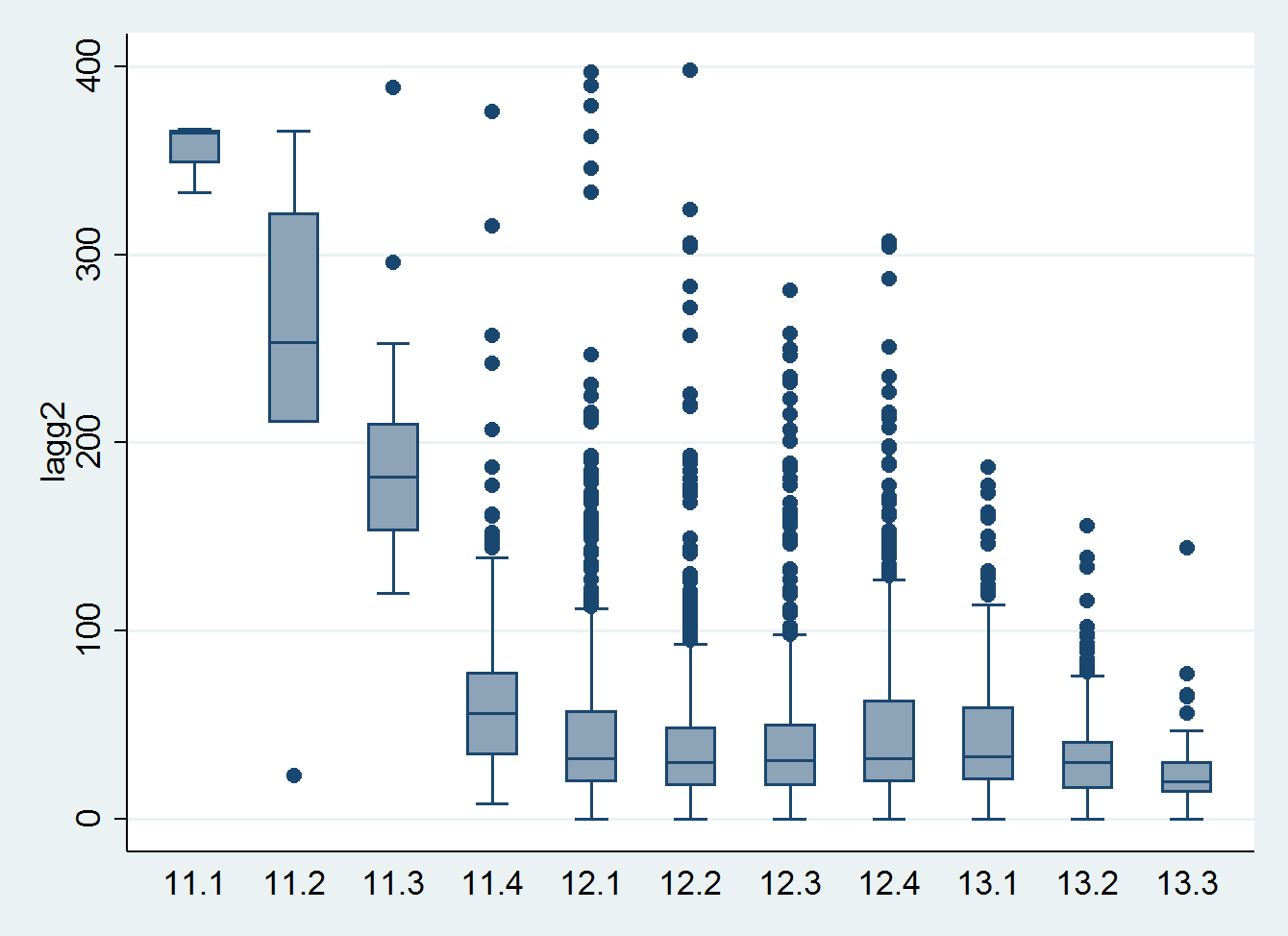


OD (days)

Seasons numbered by year and quarter of the year, so e.g. 11.3 = July-Sep 2011
