# Supplementary material for: Variations in visceral leishmaniasis burden, mortality and the pathway to care within Bihar, India
Source: Parasit Vectors. 2017 Dec 7;10:601. doi: 10.1186/s13071-017-2530-9 (PMC5719561; doi:10.1186/s13071-017-2530-9)
Supplement: Supplementary file 4 — Maps of Bihar showing burdens of identified cases in study districts for January 2012 - June 2013 at (a) district level and (b) block level. (DOCX 996 kb) [file 13071_2017_2530_MOESM4_ESM.docx]

**Additional file 4:** **Figure S2.** Maps of Bihar showing burden of identified VL cases in the 8 study districts for January 2012 - June 2013 at (a) district level and (b) block level.

**a**

**b**
