# Supplementary material for: Variations in visceral leishmaniasis burden, mortality and the pathway to care within Bihar, India
Source: Parasit Vectors. 2017 Dec 7;10:601. doi: 10.1186/s13071-017-2530-9 (PMC5719561; doi:10.1186/s13071-017-2530-9)
Supplement: Supplementary file 5 — Single-variable negative-binomial regression models for OD and OT waiting times. (DOCX 23 kb) [file 13071_2017_2530_MOESM5_ESM.docx]

**Additional file 5:** **Table S3.** Results of single-variable negative-binomial regression models for OD and OT waiting times.

|  | |  | Onset-to-diagnosis | | Onset-to-treatment | |
| --- | --- | --- | --- | --- | --- | --- |
| Factor | | Number (%) | Rate ratio (95% CI) | P-value | Rate ratio (95% CI) | P-value |
| Age | Each 1-year increase | 5375 (100) | 1.004 (1.003-1.005) | <10^-4^ | 1.005 (1.004-1.006) | <10^-4^ |
| Sex | Male | 3161 (58.77) | - | - | - | - |
|  | Female | 2218 (41.23) | 1.02 (0.97-1.06) | 0.76 | 1.02 (0.98-1.07) | 0.291 |
| District | Saharsa | 1447 (26.90) | 1.54 (1.42-1.67) | <10^-4^ | 1.54 (1.42-1.66) | <10^-4^ |
|  | E. Champ | 1214 (22.57) | 2.20 (2.02-2.39) | <10^-4^ | 2.33 (2.15-2.52) | <10^-4^ |
|  | Samastipur | 862 (16.03) | 1.66 (1.52-1.81) | <10^-4^ | 1.68 (1.55-1.84) | <10^-4^ |
|  | Gopalganj | 666 (12.38) | 2.00 (1.83-2.20) | <10^-4^ | 2.09 (1.92-2.29) | <10^-4^ |
|  | Begusarai | 459 (8.53) | - | - | - | - |
|  | Khagaria | 344 (6.40) | 1.86 (1.67-2.07) | <10^-4^ | 1.61 (1.45-1.80) | <10^-4^ |
|  | Patna | 259 (4.82) | 2.17 (1.93-2.44) | <10^-4^ | 2.03 (1.81-2.28) | <10^-4^ |
|  | W. Champ | 128 (2.38) | 3.23 (2.76-3.77) | <10^-4^ | 2.86 (2.45-3.35) | <10^-4^ |
| House  type | Kachcha | 3619 (67.29) | - | - | - | - |
|  | Semi-pucca | 1223 (22.74) | 1.03 (0.98-1.08) | 0.317 | 1.04 (0.99-1.10) | 0.097 |
|  | Pucca | 536 (9.97) | 1.03 (0.96-1.11) | 0.453 | 1.04 (0.96-1.11) | 0.338 |
| Walls | Grass+straw | 2357 (46.03) | - | - | - | - |
|  | Mud | 637 (12.44) | 0.89 (0.83-0.95) | 0.001 | 0.88 (0.82-0.94) | <10^-4^ |
|  | Mud+stone | 1017 (19.86) | 1.06 (1.00-1.12) | 0.055 | 1.06 (1.01-1.12) | 0.033 |
|  | Concrete | 1110 (21.68) | 0.99 (0.94-1.05) | 0.700 | 0.99 (0.94-1.05) | 0.771 |
| Roofing | Thatch | 2539 (49.58) | - | - | - | - |
|  | Concrete | 941 (18.38) | 1.02 (0.96-1.08) | 0.601 | 1.03 (0.97-1.09) | 0.312 |
|  | Earth tile | 1111 (21.69) | 0.95 (0.90-1.01) | 0.087 | 0.97 (0.92-1.03) | 0.308 |
|  | Asbestos | 530 (10.35) | 1.00 (0.93-1.08) | 0.915 | 1.05 (0.97-1.12) | 0.213 |
| Floor | Mud | 4842 (94.85) | - | - | - | - |
|  | Concrete | 263 (5.15) | 1.01 (0.92-1.11) | 0.847 | 1.01 (0.92-1.11) | 0.860 |
| Cattle  owner | No | 2107 (39.19) | - | - | - | - |
|  | Yes | 3269 (60.81) | 1.06 (1.01-1.10) | 0.014 | 1.04 (1.00-1.09) | 0.072 |
| Rooms | Each extra room | 5100 | 1.04 (1.02-1.05) | <10^-4^ | 1.05 (1.04-1.07) | <10^-4^ |
|  | ≤2 | 4068 (79.76) | - | - | - | - |
|  | >2 | 1032 (20.24) | 1.19 (1.13-1.25) | <10^-4^ | 1.23 (1.17-1.30) | <10^-4^ |
| Test cost | Paid | 2168 (42.54) | - | - | - | - |
|  | Free | 2928 (57.46) | 0.90 (0.86-0.94) | <10^-4^ | 0.85 (0.82-0.89) | <10^-4^ |
| Treatment  cost | Free | 3828 (80.61) | - | - | - | - |
|  | Paid | 921 (19.39) | 1.09 (1.03-1.15) | 0.003 | 1.06 (1.00-1.12) | 0.049 |
| Diagnosis centre | Public | 3242 (63.83) | - | - | - | - |
|  | Private | 1837 (36.17) | 1.11 (1.06-1.16) | <10^-4^ | 1.15 (1.10-1.20) | <10^-4^ |
| Treatment centre | Public | 4277 (85.11) | - | - | - | - |
|  | Private | 748 (14.89) | 1.04 (0.98-1.10) | 0.225 | 0.98 (0.93-1.04) | 0.550 |
| Caste | Upper | 1620 (30.48) | - | - | - | - |
|  | Lower | 3695 (69.52) | 0.90 (0.86-0.95) | <10^-4^ | 0.89 (0.85-0.93) | <10^-4^ |
| Pre-  Diagnosis  treatments | 0 | 309 (5.75) | - | - | - | - |
|  | 1 | 2004 (37.27) | 0.53 (0.47-0.59) | <10^-4^ | 0.66 (0.55-0.80) | <10^-4^ |
|  | 2 | 1715 (31.90) | 0.67 (0.60-0.75) | <10^-4^ | 0.84 (0.69-1.02) | 0.083 |
|  | 3 | 853 (15.86) | 0.93 (0.82-1.04) | 0.202 | 1.14 (0.93-1.38) | 0.207 |
|  | 4 | 496 (9.22) | 1.21 (1.07-1.37) | 0.003 | 1.51 (1.23-1.84) | <10^-4^ |
| Same-district diagnosis | No | 418 (8.49) | - | - | - | - |
|  | Yes | 4505 (91.51) | 0.88 (0.81-0.95) | 0.001 | 0.87 (0.80-0.94) | <10^-4^ |
| Same-block diagnosis | No | 2073 (43.64) | - | - | - | - |
|  | Yes | 2677 (56.36) | 0.85 (0.81-0.89) | <10^-4^ | 0.82 (0.78-0.85) | <10^-4^ |
